# Correction to: Premigratory Neural Crest Stem Cells Generate Enteric Neurons Populating the Mouse Colon and Regulating Peristalsis in Tissue-Engineered Intestine

**DOI:** 10.1093/stcltm/szad026

**Published:** 2023-05-08

This is a correction to: Huipu Yuan, Hui Hu, Rui Chen, Wenbo Mu, Liangliang Wang, Ying Li, Yuelei Chen, Xiaoyan Ding, Yongmei Xi, ShanShan Mao, Mizu Jiang, Jie Chen, Yong He, Lang Wang, Yi Dong, Jinfa Tou, Wei Chen, Premigratory neural crest stem cells generate enteric neurons populating the mouse colon and regulating peristalsis in tissue-engineered intestine, *Stem Cells Translational Medicine*, Volume 10, Issue 6, June 2021, Pages 922–938, https://doi.org/10.1002/sctm.20-0469

In the originally published version of this article, there was an error in the funding information that was presented in the second sentence of the Acknowledgments section. The sentence should have read as follows: “This research was financially supported by Xiamen’s two hundred talent Program; National Key Research and Development Program of Zhejiang, Grant/Award Number: 2020C03113; Zhejiang Provincial Natural Science Foundation of China, Grant/Award Number: LZ18C090001; National Natural Science Foundation of China, Grant/Award Number: 31171423; National Key Research and Development Program of China, Grant/Award Numbers: 2014CB541705, 2012CB967903.”

These details have been corrected only in this correction notice to preserve the published version of record.

